# DNA methylation and gene expression of immune cell markers in adolescents with chronic cannabis use: an exploratory study

**DOI:** 10.1186/s12888-024-06043-0

**Published:** 2024-10-11

**Authors:** Anne-Christine Plank, Melina Wiedmann, Sören Kuitunen-Paul, Wolfgang Wagner, Juan-Felipe Perez-Correa, Julia Franzen, Charalampos Ioannidis, Peter Mirtschink, Veit Roessner, Yulia Golub

**Affiliations:** 1 Department of Child and Adolescent Mental Health, University Hospital Erlangen, Friedrich-Alexander-Universität Erlangen-Nürnberg, Erlangen, Germany; 2 Department of Child and Adolescent Psychiatry, Faculty of Medicine, Technische Universität Dresden, Dresden, Germany; 3 Chair for Clinical Psychology and Psychotherapy, Technische Universität Chemnitz, Chemnitz, Germany; 4 Institute for Stem Cell Biology, Helmholtz-Institute for Biomedical Engineering, RWTH Aachen University Medical School, Aachen, Germany; 5 Institute for Clinical Chemistry and Laboratory Medicine, University Hospital Dresden, Dresden, Germany; 6 Department of Child and Adolescent Psychiatry, Psychosomatic and Psychotherapy, School of Medicine and Health Sciences, Carl von Ossietzky Universität Oldenburg, Oldenburg, Germany

**Keywords:** Chronic cannabis use, DNA methylation, Gene expression, Adolescents, White blood cells, B cells

## Abstract

**Background:**

Experimental studies indicate that phytocannabinoids have immune-modulatory properties. However, the effects of chronic cannabis use (CCU) in adolescents on their immune cells have been scarcely investigated to date, although CCU is increasingly observed in this age group.

**Methods:**

In this study, we analyzed DNA methylation and gene expression of immune cell markers in whole-blood samples of adolescent CCU-outpatients and non-cannabis-using (NCU) controls (*n* = 14 vs. *n* = 15, mean age = 16.1 ± 1.3 years). Site-specific DNA methylation values were used to calculate A) proportion estimates of circulating white blood cell (WBC) types and B) mean DNA methylation values of common immune cell markers (*CD4, CD8A, CD19, FCGR3A, CD14, FUT4, MPO*), whose gene expression levels were additionally determined.

**Results:**

CCU adolescents had a lower estimated proportion of B cells compared to NCU subjects. An originally observed higher proportion of granulocytes in CCU subjects, however, was attenuated when controlling for past-year tobacco use. The observed differences in mean DNA methylation and gene expression of immune cell markers were not statistically significant.

**Conclusion:**

The results of our explorative study indicate that CCU in adolescents is associated with altered levels of circulating WBCs. Further studies with larger cohorts are warranted to confirm our findings and to provide insights regarding their functional consequences.

**Supplementary Information:**

The online version contains supplementary material available at 10.1186/s12888-024-06043-0.

## Background

The consumption of cannabis has a long history marked by controversial views about its legality and risks, both regarding its application for medical purposes and as psychoactive drug. Concerning the latter, it is the most frequently used illicit substance worldwide and, as such, a major public health issue due to its negative physical and psychological outcomes [1]. Chronic cannabis use (CCU) has been, among others, linked to respiratory and vascular issues [2] and an increased risk of developing psychotic disorders [3]. Given that the use of cannabis also affects brain development, adolescents are considered to be especially vulnerable to its negative effects: CCU in adolescence has been associated with a decline in cognitive function, comprising impaired learning, memory and thinking [4, 5]. Still, a trend toward increasing societal acceptance and a less harmful view of cannabis use is observed, particularly among adolescents [6]. The lifetime use of cannabis among German adolescents has been increasing from 6.7% in 2011 to 9.3% in 2021, with 3.5% of adolescent users reporting cannabis use during the previous month and 1.6% consuming cannabis on a regular basis (defined as more than 10 times within the last 12 months) [7].

In addition, more and more countries are legalizing cannabis and cannabis-based products for recreational and medical purposes. Of the numerous chemical compounds derived from the cannabis plant (*Cannabis sativa*), the two most extensively studied phytocannabinoids are cannabidiol (CBD) and trans-Δ9-tetrahydrocannabinol (THC). THC is known to be the major psychotropic component of cannabis, limiting its therapeutic use due to adverse effects, whereas the non-psychotropic CBD is considered to have a high potential for therapeutic use. The use of medicinal cannabis (containing both THC and CBD) in the treatment of, e.g., chronic pain, chemotherapy-induced nausea and vomiting, anxiety, seizure disorders and inflammatory bowel disease [8–10] illustrates that cannabis has diverse effects on a variety of physiological processes, including modulations of the immune system. Both THC and CBD have been shown to possess immuno-modulatory and anti-inflammatory properties in vitro and in animal models (reviewed by, e.g., [11, 12]). THC primarily exerts its effects through cannabinoid receptors of the endocannabinoid system (ECS): while cannabinoid receptor 1 (CB1) is mainly localized in the central nervous system [13] and mediates the psychotropic effects of THC, cannabinoid receptor 2 (CB2) is predominantly expressed by lymphoid organs and immune cells [14], with highest mRNA levels in B cells, followed by natural killer (NK) cells, monocytes, polymorphonuclear leukocytes, CD8 + T cells and CD4 + T cells [14]. Accordingly, the ECS is considered to be a key regulator of the immune system and to modulate both innate and adaptive immune responses [15]. In contrast to THC, CBD only displays weak affinity for cannabinoid receptors [16], in spite of its impact on immune functions. However, it has been shown that CBD (and THC) can act via several further receptors (e.g., PPARγ, GPR55) and ion channels (e.g., members of the TRPA/TRPV channel families) involved in ECS signaling [15, 17]. Notably, genetic variants of genes encoding such receptors and other proteins involved in the action, metabolism and transport of cannabinoids may explain the inter-individual differences observed in the therapeutic and adverse effects of phytocannabinoids [18]. With regard to immune function, for instance, a polymorphism in the CB2 receptor gene has been linked to altered endocannabinoid immune modulation [19].

The impact of cannabinoids on the immune system is regarded as biphasic, exhibiting both stimulatory and inhibitory effects contingent on the specific type and concentration of cannabinoids involved [20]. The immunosuppressive and anti-inflammatory effects of cannabinoids have been demonstrated in numerous experimental models, both in vitro and in vivo, and are exerted via multiple mechanisms. These comprise suppression of pro-inflammatory cytokines and chemokines [21], inhibition of immune cell proliferation / activation [11, 20], induction of immune cell apoptosis [22] as well as induction of regulatory T cells and of immunosuppressive myeloid-derived suppressor cells (MDSCs) [23]. Recent studies suggest that the molecular mechanisms mediating these effects involve, among others, regulation of gene expression via epigenetic modulations [23], such as DNA methylation (i.e., the attachment of a methyl group to a cytosine base, commonly in a CpG dinucleotide context). Several studies have shown that cannabis use can impact DNA methylation, both on a genome-wide scale and of candidate genes [24, 25]. For instance, induction of MDSC by THC has been associated with altered methylation profiles of key genes involved in the differentiation and function of these immune cells [26].

In medical conditions requiring modulation or suppression of immune responses, such as autoimmune diseases or organ transplantation, the proposed immune-modulatory and anti-inflammatory properties of cannabis-derived cannabinoids could be beneficial. On the other hand, animal studies have demonstrated an association between cannabinoids and a compromised resistance to different pathogens, e.g., *Legionella pneumophila* or certain viral infections [11, 27, 28]. Although these findings implicate that particularly CCU might have adverse immunological consequences, there is only a limited number of studies with inconclusive outcomes as to whether cannabis use impairs immune competence in human subjects [29, 30]. Several studies assessing immune-related effects of cannabis use in HIV patients have suggested that cannabis does not adversely affect their immune status [31–33]. In adult individuals without an infectious disease, there is limited evidence supporting a link between regular exposure to cannabis and a decrease in the production of several factors mediating inflammation [29]. However, such associations were not confirmed for CRP (C-reactive protein, a marker of inflammation) in cannabis-using adolescents and young adults [34, 35]. The few studies to date that have examined the numbers of circulating white blood cells (WBCs) in cannabis users also provide mixed results [25, 36–38]. In summary, more research is urgently needed to uncover the effects of CCU on immune function – especially in adolescence, since the vulnerability to the negative effects of cannabis is increased during this developmental period [5]. In the present exploratory study, we therefore aimed to investigate the association of CCU with leukocyte measures, including DNA methylation and expression levels of different immune cell markers, in an adolescent outpatient cohort. We hypothesize that CCU is linked to alterations in leukocyte proportions and the expression of associated immune cell markers in adolescent users.

## Material and methods

### Study design and participants

The study, part of a larger project at C. G. Carus University Hospital, evaluated a group-based intervention program for adolescents with chronic substance use. Data and sample collection for the current analyses was integrated into routine diagnostic procedures. Prior to their participation in the study, all adolescents and their legal guardians provided written informed consent/assent. The study was approved by the local ethics committee of the Medical Faculty of the TU Dresden (EK 66022018) and conducted in accordance with the Declaration of Helsinki. For further information see Wiedmann et al. (2022) [24].

**Fig. 1:**
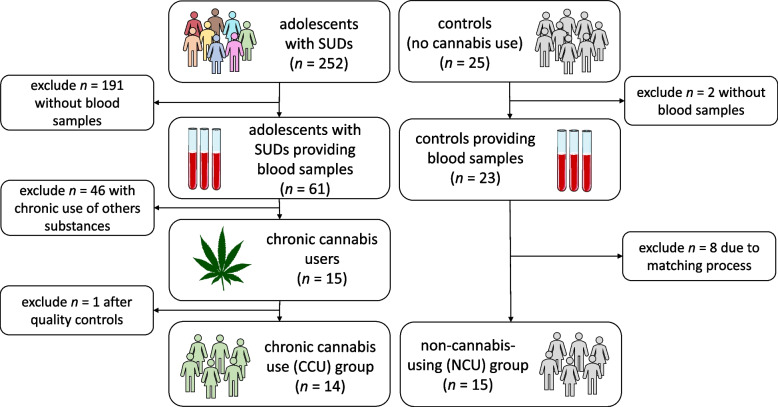
Flow chart* of sample composition for the CCU (chronic cannabis use) group and the control group (non-cannabis-using, NCU). *The Figure was partly generated using Servier Medical Art, provided by Servier, licensed under a Creative Commons Attribution 3.0 unported license

Of *n* = 252 adolescents with substance use disorders (SUD), *n* = 61 provided blood samples. Of these, *n* = 46 adolescents were excluded because of either use of illicit substances other than cannabis during the past month / past 12 months (more than weekly use), or due to medication intake. In addition, *n* = 1 adolescent had to be excluded due to technical reasons (insufficient sample quality). The final CCU group comprised *n* = 14 adolescents with CCU (shown in Fig. 1). *N* = 25 age- and gender-matched adolescents with no self-reported lifetime use of cannabis or any other illicit substance were recruited from other psychiatric departments of the clinic and via local advertisement. *N* = 23 agreed to provide blood samples. The non-cannabis-using (NCU) control group was further matched to the CCU group with regard to alcohol and tobacco use, depressive symptoms (BDI-II scores) and other co-occurring psychiatric disorders (psychotic disorders, mood disorders, anxiety disorders, conduct disorders, obsessive compulsive disorders, posttraumatic stress disorders, eating disorders and tic disorders). The final NCU group comprised* n* = 15 adolescents (shown in Fig. 1).

### Substance use

We conducted interviews and qualitative urine tests to assess the use of various substances such as tobacco, alcohol, cannabis, 3,4-methylenedioxymethamphetamine (MDMA), amphetamine, methamphetamine, cocaine, hallucinogens, ketamine, opiates and others. The extent of substance use was measured based on average quantity and frequency of use in the past 12 months. CCU was defined as weekly cannabis use during the past 12 months, accompanied by related problems. Using criteria from ICD-10, cannabis dependence (F12.2) and harmful use (F12.1) were identified with 28.6% meeting the criteria for cannabis dependence, and 71.4% for harmful use (Table 1), see also Wiedmann et al. (2022) [24].

**Table 1:** Characteristics of demographics, substance use and co-occurring disorders of the sample

	**NCU group** **(*n* = 15)**	**CCU group** **(*n* = 14)**	**Group comparison**			
	*M* (*SD*) / *N* (%)	*M* (SD) / *N* (%)	*t / Χ*^*2*^*/ U*	*df*	*p*	*d / φ / r*
Cannabis use^a^	0.0 (0.0)	21.0 (10.9)	-2.90^d^	13	.012*	-1.12
Alcohol consumption^a^	0.3 (0.4)	5.1 (8.5)	-1.81^d^	10.02	.100	-0.81
Smokers	3 (20%)	14 (100%)	19.11^e^	1	< .001*	.81
Tobacco use^a^	4.1 (10.5)	24.9 (8.8)	-2.27^d^	18.91	.035*	-0.86
Sex (males)	11 (73.3%)	11 (78.6%)	.11^e^	1	.742	-.06
Age (years)	15.4 (1.8)	16.1 (1.3)	-1.31^d^	27.00	.201	-0.49
BDI-II Score	11.1 (11.9)	10.0 (8.9)	0.27^d^	26.00	.792	0.10
Cannabis dependence (ICD-10)	0 (0%)	4 (28.6%)	16.35^e^	1	< .001*	.75
Cannabis harmful use (ICD-10)	0 (0%)	10 (71.4%)	4.97^e^	1	.026*	.41
Co-occurring psychiatric disorders^b^	0.6 (0.7)^c^	1.2 (1.3)^c^	75.00^f^	-	.404	0.16
Common cold (no fever) at time of blood sampling	3 (20%)	2 (14%)	.17^e^	1	.684	-.08
BMI (age percentile)	50.20 (37.53)	52.07 (29.02)	-0.149^d^	27	.882	-.06

*NCU* non-cannabis-using, *CCU* chronic cannabis use, *BDI-II* Beck’s Depression Inventory version 2, *BMI* body mass index. *a* average days per month during past year. *b* *DSM*-5 average number, excluding Substance Use Disorders* c,* *n* = 1 missing due to missed appointment. Statistics *d,* independent *t*-tests. *e*, Chi-Square tests. *f,* Mann–Whitney *U* test. *p < .05

### Diagnosis of psychiatric disorders

The assessment of co-occurring psychiatric disorders was conducted using the German Diagnostic Interview of Psychiatric Disorders [DIPS [39, 40]]. This structured interview follows the DSM-5 criteria and focuses on evaluating the presence of psychiatric disorders in adolescents. The identified disorders were categorized into specific classes as follows: SUD with ICD-10 codes F1X.1 and F1X.2, Depressive Disorders (F32.X, F33.X), Anxiety Disorders (F40.X, F41.X), Posttraumatic Stress Disorder (PTSD; F43.1), Hyperkinetic Disorders (F90.X, F98.8), Conduct Disorders (F90.1, F91.X, and F92.X), Personality Disorders (F93.8 and F43.X except F43.1), and Psychotic Disorders (F1X.5, F1X.7, F30.2, F2X.X).

### DNA methylation analysis

DNA methylation data investigated in the present study was derived from a genome-wide DNA methylation analysis. Fasting venous blood samples were collected in the morning and stored at –80°C until further processing. All adolescents reported at least 24h abstinence from cannabis at the time of blood drawing. Genomic DNA was isolated from 1.8 – 2.0 ml EDTA whole blood samples using the Qiagen Blood Mini kit (Qiagen, Hilden, Germany) and 1.2 µg were sent to Life & Brain GmbH (Bonn, Germany) for bisulfite conversion and subsequent analysis using the Infinium MethylationEPIC BeadChip (Illumina, San Diego, CA, USA). DNA methylation analysis was performed by Cygenia GmbH (Aachen, Germany). Beta values at approximately 850,000 CpGs were calculated after quantile normalization (using the R package minfi [41]), which range from 0 to 1 and reflect DNA methylation levels for each CpG site. To ensure data quality, various quality control measures were implemented, including probe intensities, beta value distributions, unsupervised clustering, and age/gender predictions.

#### Quantification of white blood cells

In order to estimate the proportions of WBC types (B cells, CD8 + T cells, CD4 + T cells, NK cells, granulocytes and monocytes) in each unfractionated whole blood sample, methylation data was statistically analyzed using the Houseman method [42, 43] (performed by Cygenia GmbH, Aachen, Germany).

#### DNA methylation of immune cell marker genes

The immune cell markers selected for CpG site methylation analyses are displayed in Table 2. Methylation levels of CpG sites associated with the target genes were derived from the whole-genome methylation datasets. To assess CCU-dependent differences in methylation levels, the mean beta values of all CpG sites assessed per target gene were calculated. Further information on individual CpG sites is provided in Table S1.

**Table 2:** Immune cell markers selected for analysis

marker	immune cell type
*CD19*	B cells
*CD8A*	cytotoxic T cells
*CD4*	T helper/regulatory cells
*FCGR3A* (CD16)	NK cells
*CD14*	monocytes
*FUT4* (CD15)	granulocytes
*MPO*	neutrophils

*FUT4* fucosyltransferase 4, catalyzes the synthesis of CD15, a marker for granulocytes, i.e. neutrophils, eosinophils, basophils, *MPO* myeloperoxidase, released by activated neutrophils

### Gene expression analysis

RNA extraction from whole blood samples stored at -80°C was performed as described by Kim et al. [44] (see Supplementary Information for details). Reverse transcription was performed with the iScript cDNA Synthesis kit (Bio-Rad, Hercules, CA, USA) and cDNA was analyzed by qPCR using the SsoFast Eva Green Supermix (Bio-Rad, Hercules, CA, USA), a CFX384 real-time System C1000 Thermal Cycler (Bio-Rad, Hercules, CA, USA) and the Bio-Rad CFX Manager 3.1 software. Target gene Ct values were normalized to *18S*. Primers used for gene expression analyses of immune cell markers are listed in Supplementary Table S2.

### Statistical analyses

Statistical analyses were performed using IBM SPSS Statistics Version 28.0.1.1 (IBM Corp., Armonk, NY, USA). Descriptive data are reported as means (*M*) and the standard deviation (*SD*). Outliers were defined as values > 2.7 *SD*s from *M* and were excluded from analyses. Kolmogorov–Smirnov tests were conducted to verify normal distribution of the data (results are displayed in the Supplementary Information). For group comparisons, Chi-Square tests and independent *t*-tests were performed. In case of significantly different variances, as indicated by Levene’s test (*p* ≤ 0.05), *t* values, *p* values and degrees of freedom (*df*) were adjusted. When data was not normally distributed, groups were further compared in a non-parametric test (Mann–Whitney *U*), the results of which are only reported in case of deviating outcomes regarding the level of significance. Effect sizes are reported as *φ* / Cohen’s *d* / *r* and interpreted according to Cohen [45] as small (0.20 ≤ |*d*| ≤ 0.49 / 0.10 ≤ |r or *φ*| ≤ 0.29), medium (0.50 ≤ |*d*|≤ 0.79 / 0.30 ≤ |r or *φ*| ≤ 0.49) or large (|*d *|> 0.79 / |r or *φ*| > 0.49). In case of significant group differences, a potential confounding effect of tobacco exposure was addressed by performing MANCOVA (Multivariate Analyses of Covariance) with past year tobacco exposure as control variable, estimated WBC proportion per cell type / target gene methylation / target gene expression as dependent variables and group membership as the predictor variable, reporting *F*-values, *p*-values and partial eta squared (η^2^_part_) for effect size estimations (η^2^_part_ ≥ 0.01 was interpreted as small effect, η^2^_part_ ≥ 0.06 as a medium effect size and η^2^_part_ ≥ 0.14 as a strong effect [45]). The level of significance was defined as *p* < 0.05 (two-tailed); *p* < 0.10 was interpreted as a trend to significance. Since estimated cell type proportions are interrelated, we used the False Discovery Rate (Benjamini–Hochberg correction) to control for alpha inflation due to multiple testing. However, since our study was exploratory, with a small sample size, we opted not to correct for multiple testing regarding immune cell marker DNA methylation and gene expression. This allows for a broader exploration of the data and facilitates the generation of hypotheses and novel insights.

## Results

### Descriptive data and pre-analyses

The CCU group had a mean age of 16.1 (*SD* = 1.3) years and included 79% male adolescents (*n* = 11). Since matching controls were selected, the groups did not differ significantly in age, gender, body mass index (BMI), somatic and psychiatric disorders. None of the participants was suffering from an acute infection or taking medication at the time of examination and blood collection. Despite the selection of the best-fitting NCU controls, the number of smokers and frequency of tobacco use in the past 12 months was significantly higher in the CCU group (Table 1).

### Estimated proportions of leukocyte subsets

**Fig. 2:**
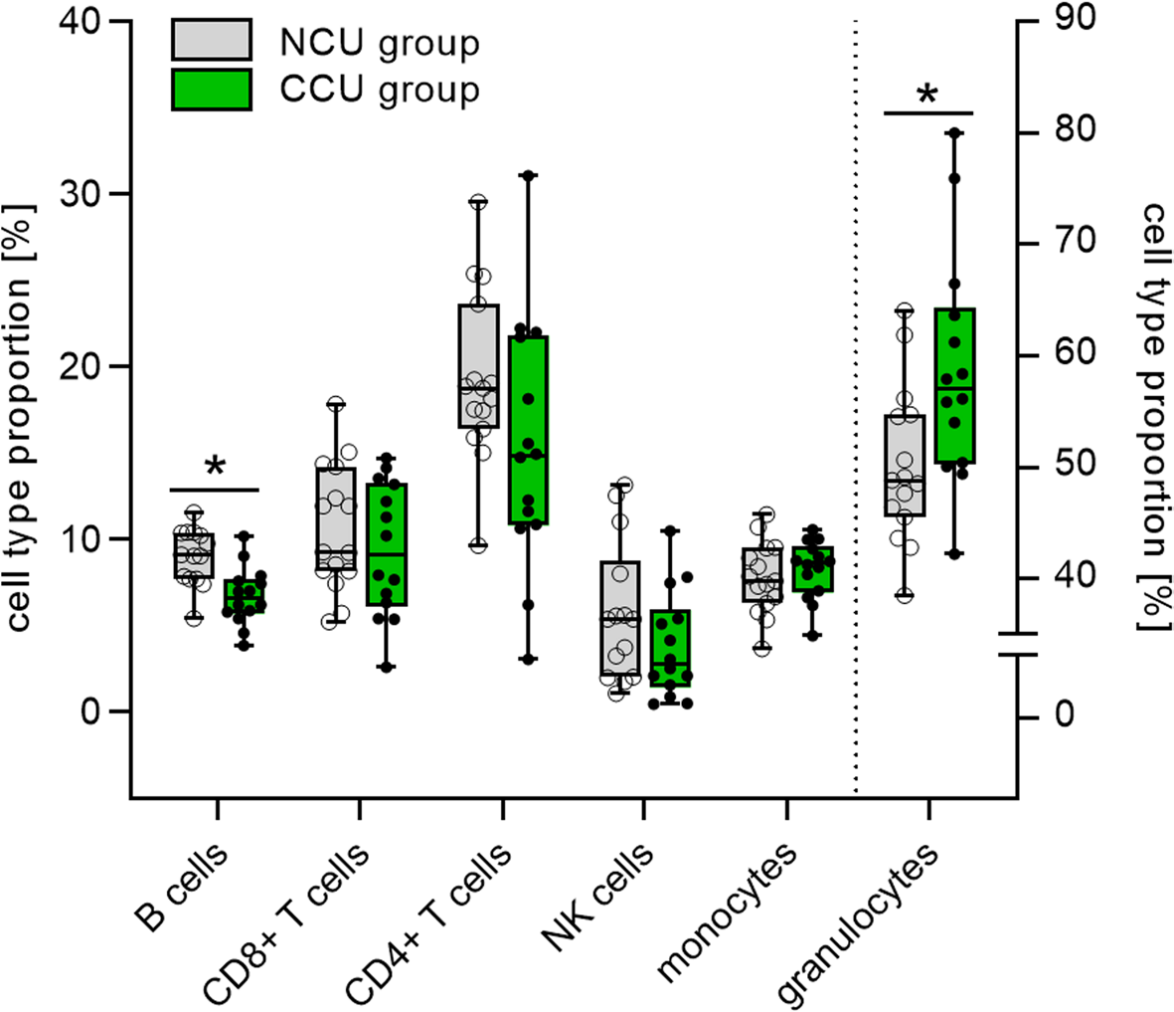
Estimated proportions of leukocyte subsets in unfractionated whole blood samples of the non-cannabis-using (NCU, *n* = 15 / B and NK cells: *n* = 14) and the chronic cannabis use (CCU, *n* = 14) group. Box-whiskers plot, whiskers: minimum to maximum. Independent *t*-tests, *p < .05

The relative quantification of immune cell type proportions revealed a significantly smaller fraction of B cells in CCU adolescents compared to the NCU group (Fig. 2 and Table S3). This difference remained significant after controlling for multiple testing and past year tobacco use (*F*(25) = 10.07, η^2^_part_ = 0.30, *p*_*adjusted*_ = 0.08). We also found a higher proportion of granulocytes in the CCU group (Fig. 2 and Table S3); however, this difference was attenuated when accounting for past year tobacco use (*F*(25) = 1.55, η^2^_part_ = 0.06, *p*_*adjusted*_ = 0.226).

### DNA methylation of immune cell marker genes

In order to substantiate our findings regarding CCU-associated differences in WBC proportions, we analyzed methylation levels of CpG sites associated with common immune cell marker genes. Before controlling for past year tobacco use, comparisons via *t*-tests revealed significant differences in mean *CD19*-, *CD4*-, *FUT4*- and *MPO-*associated DNA methylation, with the CCU group showing higher *CD19*- and *CD4-*associated methylation and lower *FUT4* and *MPO* methylation levels (Table 3). The trend to lower *FCGR3A* methylation was not statistically significant. CCU and NCU samples did not differ in *CD8A-* and *CD14-*associated DNA methylation [Table 3; see the supplementary material for heatmaps visualizing the individual methylation levels per CpG site for *CD19* and *FUT4* (Fig S1A and B) and the mean individual methylation levels for all target genes (Fig. S2)]. When controlling for past year tobacco exposure, all significant differences were attenuated (*CD19*: *F*(26) = 3.51; η^2^_part_ = 0.13; *p* = 0.073; *CD4: F*(26) = 1.34; η^2^_part_ = 0.05; *p* = 0.259; *FUT4*: *F*(26) = 0.50; η^2^_part_ = 0.02; *p* = 0.487; *MPO*: *F*(26) = 0.60; η^2^_part_ = 0.02; *p* = 0.446). However, the effect size reported for *CD19* (η^2^_part_ = 0.13) remained at medium extent.

**Table 3:** Mean methylation levels of CpG sites of *k* = 7 target genes in NCU (*n* = 15) and CCU (*n* = 14) adolescents

Target gene	NCU group (*n* = 15)		CCU group (*n* = 14)		Group comparison		
	*M*	*SD*	*M*	*SD*	*t (df)*	*p*	*d*
*CD19*	0.585	0.005	0.590	0.008	-2.12 (27)	.043*	-0.788
*CD8A*	0.428	0.010	0.432	0.011	-1.10 (27)	.281	-0.409
*CD4*	0.747	0.009	0.758	0.014	-2.42 (27)	.022*	-0.900
*FCGR3A*	0.557	0.006	0.552	0.008	1.84 (27)	.076	0.685
*CD14*	0.270	0.015	0.271	0.017	-0.25 (27)	.806	-0.092
*FUT4*	0.365	0.019	0.341	0.035	2.26 (27)	.032*	0.839
*MPO*	0.561	0.025	0.523	0.053	2.47 (18.3)	.024*	0.939

*NCU* non-cannabis-using, *CCU* chronic cannabis use. Statistics, Independent *t*-tests, *p < .05

### Immune cell marker gene expression

Results of independent *t*-tests comparing group means of relative target gene expression are displayed in Table 4. On average, the CCU group showed a non-significant trend to lower expression of *CD19* and to higher expression of *CD14* and *FUT4* than the NCU group (*CD14*: *U* = 141, *p* = 0.045; *FUT4*: *U* = 148, *p* = 0.020). However, controlling for past year tobacco use attenuated the significant difference in *CD14* and *FUT4* expression (*CD14*: *F*(25) = 0.85; η^2^_part_ = 0.04; *p* = 0.366; *FUT4*: *F*(25) = 0.82; η^2^_part_ = 0.03; *p* = 0.376). Expression levels of *CD8A, CD4, FCGR3A* and *MPO* did not differ between the CCU and the NCU group (Table 4).

**Table 4:** Relative expression levels of *k* = 7 target genes in NCU (*n* = 15) and CCU (*n* = 13) adolescents

Target gene	NCU group (*n* = 15)		CCU group (*n* = 13)		Group comparison			
	*M*	*SD*	*M*	*SD*	*t*	*df*	*p*	*d*
*CD19*^*1*^	1.26E-06	5.74E-07	9.11E-07	3.80E-07	1.80	25	.083	0.698
*CD8A*^*1*^	4.33E-06	1.55E-06	4.16E-06	1.37E-06	0.30	25	.769	0.115
*CD4*	1.67E-05	5.78E-06	2.66E-05	2.69E-05	-1.30	12.97	.215	-0.529
*FCGR3A*	1.30E-06	6.68E-07	1.46E-06	8.18E-07	-0.57	26	.576	-0.214
*CD14*	1.21E-04	8.41E-05	2.56E-04	2.32E-04	-2.00	14.73	.064	-0.803
*FUT4*	3.28E-06	2.36E-06	6.43E-06	5.20E-06	-2.01	16.22	.061	-0.801
*MPO*	6.57E-08	4.49E-08	1.23E-07	1.27E-07	-1.54	14.60	.145	-0.617

Due to insufficient material obtained from one *CCU* participant, analyses were performed for *n* = 13 *CCU* adolescents. ^1^ *n* = 1 sample of the *CCU* group was identified as outlier, the respective value was excluded from analysis. *NCU* non-cannabis-using, *CCU* chronic cannabis use. Statistics: Independent *t*-tests

## Discussion

A growing body of literature has shown that during adolescence, the developing body and brain are especially sensitive to the effects of cannabis use [5]. Experimental studies further provide evidence that cannabinoids have immune-modulatory and anti-inflammatory properties; however, little literature exists regarding the effect of cannabis use on circulating immune cells, particularly in adolescents. Our exploratory study examined the association between CCU and leukocyte characteristics in an adolescent outpatient cohort with self-reported CCU, defined as at least weekly use of cannabis during the past 12 months. Since our study was conducted with a clinical sample, it is important to note that adolescent patients who abuse cannabis often concurrently smoke tobacco [46]. Given this common co-occurrence, it becomes challenging to disentangle the distinct effects of cannabis and tobacco on immune markers in this cohort. To address this issue, we incorporated tobacco use as a covariate in our statistical models, thereby controlling for its potential confounding effect. Consequently, the originally observed higher proportion of granulocytes in CCU subjects was attenuated when controlling for past-year tobacco use. Furthermore, the observed differences in mean DNA methylation and gene expression of immune cell markers were not statistically significant. Importantly, the methylation-based quantification of immune cell type proportions revealed a significantly lower proportion of B cells in the CCU group. We also observed a trend to reduced gene expression of the B cell marker CD19. Although not statistically significant, a reduced expression would match the differences observed in the mean CpG site methylation levels of *CD19,* since increased methylation is commonly associated with reduced gene expression [47]*.* Previous research investigating WBC counts in cannabis users has yielded mixed results: one study observed a significant decrease only in the number NK cells, but not for T and B cells as compared to controls [37]. In the context of their methylome-wide association study (MWAS) investigating the effects of cannabis use on the methylome, Clark et al. [25] report slightly decreased B cell levels in adolescents with problematic cannabis use compared to non-problematic users, yet equal levels of T cells, monocytes and granulocytes. El-Gohary and Eid, who analyzed leukocyte profiles of young adults using bhang (an edible form of cannabis), found decreased numbers of NK, T and B cells [36]. In our study, we could not detect CCU-associated differences in the proportion of NK cells and CD4+ T cells. Still, our observation of a significant reduction in B cells, which are the central element of humoral immunity and a major component of the adaptive immune system, is in line with previous findings.

**Fig. 3:**
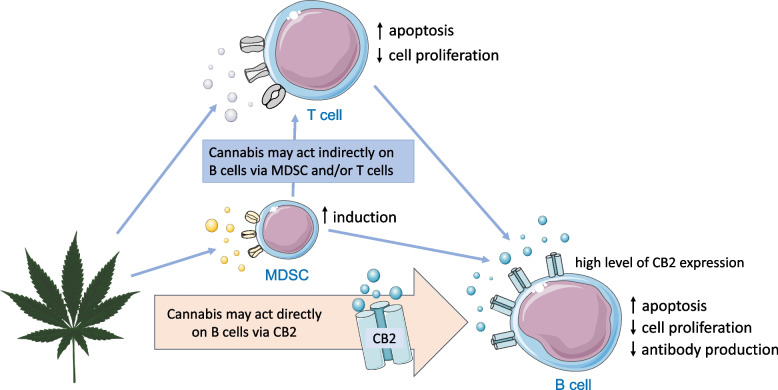
Schematic illustration* of hypothesized CCU effects on B cells. Studies investigating the effect of cannabinoids on B cells have provided evidence for enhanced apoptosis, decreased cell proliferation and reduced antibody production. B cells express the highest amount of CB2 on their cell surface as compared to other leukocyte subtypes, implying that the underlying mechanism of action may involve a CB2-mediated reduction of B-cell proliferation or survival. However, cannabinoids could also act on B lymphocytes indirectly via modulation of other immune cells, such as MDSC or T cells. MDSC: myeloid-derived suppressor cell; CB2: Cannabinoid receptor 2. *The Figure was partly generated using Servier Medical Art, provided by Servier, licensed under a Creative Commons Attribution 3.0 unported license, and illustrations provided by pixabay (https://pixabay.com/de/vectors/marihuana-blatt-gr%c3%bcn-topf-cannabis-34178/)

Although differential effects of dose (low vs. high) and origin of the acting cannabinoid (endo- vs. phyto- or synthetic) on B cell proliferation (stimulation vs. inhibition) are discussed [20], experimental studies investigating the effect of cannabinoids on B cells have provided evidence for enhanced apoptosis [23], decreased cell proliferation and reduced antibody production (reviewed by [20]) (Fig. 3). B cells express the highest amount of CB2 on their cell surface as compared to other leukocyte subtypes [14], implying that the underlying mechanism of action may involve a CB2-mediated reduction of B-cell proliferation or survival. The levels of CB2 mRNA in peripheral blood mononuclear cells (PBMCs) of chronic cannabis users have been found to be elevated [48], and CB2 mRNA levels to remain increased in PBMCs even after abstaining from cannabis for ≥ 6 months [49]. Yet there is still a paucity of studies identifying B cells as direct targets of the ECS [11]; cannabinoids could also act on B lymphocytes indirectly via other immune cells, such as MDSC or T cells [23], and through receptors other than CB2. A potential mechanistic link is suggested by Clark et al. [25]: based on their MWAS-results, they hypothesize that problematic cannabis use may alter methylation of genes relevant for DNA repair in B cells, with potential downstream effects on immune functioning. Such effects may involve impaired humoral immune responses to pathogens along with reduced vaccination-induced immunity [15], yet more research is needed to elucidate the mechanisms and clinical outcome of CCU-related effects on the adaptive immune system.

Other than B cells, granulocytes (or polymorphonuclear leukocytes) are effectors of the innate immune system. They comprise eosinophils, basophils and neutrophils, which are the most abundant subtype of circulating WBC (50–70%) and continuously generated in the bone marrow from myeloid precursors. Neutrophils are typically the first leukocytes to be recruited to injured or infected tissue, capable of eliminating pathogens by multiple mechanisms [50]. Our CCU group displayed an increased proportion of circulating granulocytes, along with lower mean CpG site methylation and increased expression of *FUT4* (encoding the enzyme fucosyltransferase 4, which catalyzes the synthesis of the granulocyte marker CD15) and of *MPO*, encoding myeloperoxidase, which is released by activated neutrophils at the site of infection [50]. We suggest that the low and CCU-independent *MPO* expression levels measured in our whole blood samples may arise from the fact that the neutrophil granules containing MPO are formed during their maturation in the bone marrow [50].

Our finding of an increased fraction of granulocytes in adolescents with CCU is consistent with results obtained from an adult sample of the US National Health and Nutrition Examination Survey, where a modest association between heavy cannabis use and increased WBC count, primarily elevated neutrophils, was detected [38]. The author suggests that this increase may be related to the inflammatory effects of combustion by-products. This aligns with our sample, where the most common mode of cannabis use is smoking, akin to tobacco cigarette smokers who have shown elevated WBC [51, 52]. The adolescents in our CCU group did not differ from the NCU group in terms of their physical health status, so that acute inflammation or disease can be excluded as a reason for the differences in granulocyte proportions; however, the CCU group did use significantly more tobacco than the NCU group. Although in the study by Alshaarawy [38], the differences in neutrophil counts remained significant after adjustment for multiple variables, including BMI, alcohol drinking and tobacco smoking, accounting for past year tobacco use attenuated the differences in granulocyte proportions and *FUT4* / *MPO* methylation levels in our sample. Nonetheless, CBD has been shown to exert direct effects on neutrophils by inhibiting their migration to sites of infection [53]. It should also be noted that chronic heavy smoking of cannabis has been associated with increased symptoms of chronic bronchitis, and CCU is suggested to have different effects on lung function than tobacco smoking [54, 55]. To determine the effects of cannabis vs. tobacco smoking on immune cells, future studies should try to adapt their sample accordingly, e.g. by including a further group of tobacco-only smoking participants, and investigating a larger sample size, which is one of the limitations of our exploratory study.

### Limitations

Due to the small sample size, we did not perform further covariate and multiple testing adjustments. However, our small sample size resulted from a strict selection of the CCU cohort, since we carefully controlled for other confounding variables, such as comorbid substance use that is often neglected in studies, and we applied careful matching procedures. Besides, the composition of leukocyte subsets is usually measured by flow cytometry. However, since such measurements were not feasible with the frozen blood samples of this study, we utilized the epigenetic markers, which have proven to have high correlation with conventional blood counts [56, 57]. We are also aware that assessing WBC proportions and the methylation / expression of selected target genes is narrow in scope with regard to the complexity of immune system components and function. Yet considering the fact that a paucity of studies investigated the effect of cannabis use on WBC in adolescents to date, we believe that our results are a valuable contribution that encourage future studies. The latter should investigate more specific leukocyte subsets, such as CD4^+^-CD25^+^ regulatory T cells, and evaluate measures of immune cell functionality, e.g. enzyme activities, levels of antibodies, cytokines and further markers of inflammation. For instance, Ferguson et al. [35] utilized data of the National Longitudinal Study of Adolescent to Adult Health to investigate the relationship of marijuana use and inflammation as measured by CRP levels, concluding that marijuana use does not confer an anti-inflammatory effect and recency of use is not relevant. Costello et al. [34] even found support for a pro-inflammatory effect of marijuana on CRP in adolescents.

These and our results illustrate that more studies providing a comprehensive evaluation of different immune cell types and their functions is needed to fill the current knowledge gap as to whether, how, and to what extent CCU affects immune competence in adolescent cannabis users. Moreover, future studies should consider a longitudinal design to examine intra-individual immunological changes, e.g. analyze adolescents before and after achieving abstinence from cannabis. Eventually, such data will allow to draw causal conclusions, which we cannot yet derive from our present results.

## Conclusion

The results of our explorative study suggest that CCU in adolescents is associated with altered levels of circulating WBCs, especially a lower proportion of B cells, and possibly linked to epigenetic changes. Further studies with larger sample sizes are warranted to confirm our findings and to provide insights regarding functional and clinical consequences of CCU-associated changes in immune cell profiles.

## Supplementary Information

Supplementary Material 1.

## Data Availability

The data that support the findings of this study are not publicly available due to their containing information that could compromise the privacy of research participants, but are available from Y.G. on reasonable request.

## Abbreviations

CB1 / CB2: Cannabinoid receptor 1 / cannabinoid receptor 2
CBD: Cannabidiol
CCU: Chronic cannabis use
CRP: C-reactive protein
ECS: Endocannabinoid system
MDMA: 3,4-Methylenedioxymethamphetamine
MDSC: Myeloid-derived suppressor cells
MWAS: Methylome-wide association study
NCU: Non-cannabis-using
NK cells: Natural killer cells
PBMC: Peripheral blood mononuclear cell
SUD: Substance use disorder
THC: Trans-Δ9-tetrahydrocannabinol
WBC: White blood cell
