# Supplementary material for: DNA methylation and gene expression of immune cell markers in adolescents with chronic cannabis use: an exploratory study
Source: BMC Psychiatry. 2024 Oct 11;24:676. doi: 10.1186/s12888-024-06043-0 (PMC11468367; doi:10.1186/s12888-024-06043-0)
Supplement: Supplementary file 1 — Supplementary Material 1. [file 12888_2024_6043_MOESM1_ESM.docx]

**DNA methylation and gene expression of immune cell markers in adolescents with chronic cannabis use: an exploratory study - Supplementary Information**

**Table S1.** DNA methylation of immune cell marker genes - characteristics of individual CpG sites

| **CpG** | **RNA** | **Gene** | **Chromo-some** | **POS** | **located in reg. region** | **GHID^1^** | **GH type** | **start^2^** | **end^2^** | |
| --- | --- | --- | --- | --- | --- | --- | --- | --- | --- | --- |
| cg01477015 | CD19 | *CD19* | 16 | 28948319 | 1 | GH16J028936 | Promoter/Enhancer | 28947121 | 28948475 | |
| cg01758575 | CD19 | *CD19* | 16 | 28943288 | 1 | GH16J028930 | Promoter/Enhancer | 28941323 | 28944841 | |
| cg03660502 | CD19 | *CD19* | 16 | 28948092 | 1 | GH16J028936 | Promoter/Enhancer | 28947121 | 28948475 | |
| cg05409463 | CD19 | *CD19* | 16 | 28946741 | 0 |  |  |  |  | |
| cg05718426 | CD19 | *CD19* | 16 | 28949128 | 0 |  |  |  |  | |
| cg07322144 | CD19 | *CD19* | 16 | 28948179 | 1 | GH16J028936 | Promoter/Enhancer | 28947121 | 28948475 | |
| cg09989938 | CD19 | *CD19* | 16 | 28944403 | 1 | GH16J028930 | Promoter/Enhancer | 28941323 | 28944841 | |
| cg11691515 | CD19 | *CD19* | 16 | 28946150 | 0 |  |  |  |  | |
| cg14102807 | CD19 | *CD19* | 16 | 28943677 | 1 | GH16J028930 | Promoter/Enhancer | 28941323 | 28944841 | |
| cg17248408 | CD19 | *CD19* | 16 | 28944243 | 1 | GH16J028930 | Promoter/Enhancer | 28941323 | 28944841 | |
| cg17532822 | CD19 | *CD19* | 16 | 28948046 | 1 | GH16J028936 | Promoter/Enhancer | 28947121 | 28948475 | |
| cg22742914 | CD19 | *CD19* | 16 | 28948113 | 1 | GH16J028936 | Promoter/Enhancer | 28947121 | 28948475 | |
| cg24900963 | CD19 | *CD19* | 16 | 28948266 | 1 | GH16J028936 | Promoter/Enhancer | 28947121 | 28948475 | |
| cg00366435 | CD4 | *CD4* | 12 | 6896105 | 1 | GH12J006784 | Promoter/Enhancer | 6893414 | 6896747 | |
| cg02540045 | CD4 | *CD4* | 12 | 6924827 | 1 | GH12J006815 | Enhancer | 6924767 | 6926166 | |
| cg03795245 | CD4 | *CD4* | 12 | 6929671 | 1 | GH12J006818 | Promoter/Enhancer | 6927166 | 6933000 | |
| cg03855955 | CD4 | *CD4* | 12 | 6900351 | 1 | GH12J006788 | Promoter/Enhancer | 6897366 | 6902967 | |
| cg05044173 | CD4 | *CD4* | 12 | 6898500 | 1 | GH12J006788 | Promoter/Enhancer | 6897366 | 6902967 | |
| cg06370542 | CD4 | *CD4* | 12 | 6923187 | 0 |  |  |  |  | |
| cg06624527 | CD4 | *CD4* | 12 | 6898647 | 1 | GH12J006788 | Promoter/Enhancer | 6897366 | 6902967 | |
| cg08349804 | CD4 | *CD4* | 12 | 6929591 | 1 | GH12J006818 | Promoter/Enhancer | 6927166 | 6933000 | |
| cg10266328 | CD4 | *CD4* | 12 | 6924908 | 1 | GH12J006815 | Enhancer | 6924767 | 6926166 | |
| cg10581137 | CD4 | *CD4* | 12 | 6926283 | 0 |  |  |  |  | |
| cg00219921 | CD8 | *CD8A* | 2 | 87012810 | 1 | GH02J086783 | Promoter/Enhancer | 87009725 | 87022878 | |
| cg00670742 | CD8 | *CD8A* | 2 | 87034831 | 1 | GH02J086805 | Promoter/Enhancer | 87032335 | 87037723 | |
| cg00916536 | CD8 | *CD8A* | 2 | 87017419 | 1 | GH02J086783 | Promoter/Enhancer | 87009725 | 87022878 | |
| cg02170525 | CD8 | *CD8A* | 2 | 87018382 | 1 | GH02J086783 | Promoter/Enhancer | 87009725 | 87022878 | |
| cg03196485 | CD8 | *CD8A* | 2 | 87021117 | 1 | GH02J086783 | Promoter/Enhancer | 87009725 | 87022878 | |
| cg03318654 | CD8 | *CD8A* | 2 | 87034200 | 1 | GH02J086805 | Promoter/Enhancer | 87032335 | 87037723 | |
| cg07152196 | CD8 | *CD8A* | 2 | 87016436 | 1 | GH02J086783 | Promoter/Enhancer | 87009725 | 87022878 | |
| cg08506127 | CD8 | *CD8A* | 2 | 87018958 | 1 | GH02J086783 | Promoter/Enhancer | 87009725 | 87022878 | |
| cg09046939 | CD8 | *CD8A* | 2 | 87017591 | 1 | GH02J086783 | Promoter/Enhancer | 87009725 | 87022878 | |
| cg09318840 | CD8 | *CD8A* | 2 | 87018944 | 1 | GH02J086783 | Promoter/Enhancer | 87009725 | 87022878 | |
| cg09849552 | CD8 | *CD8A* | 2 | 87019492 | 1 | GH02J086783 | Promoter/Enhancer | 87009725 | 87022878 | |
| cg11423206 | CD8 | *CD8A* | 2 | 87014979 | 1 | GH02J086783 | Promoter/Enhancer | 87009725 | 87022878 | |
| cg12344862 | CD8 | *CD8A* | 2 | 87019846 | 1 | GH02J086783 | Promoter/Enhancer | 87009725 | 87022878 | |
| cg12653796 | CD8 | *CD8A* | 2 | 87016720 | 1 | GH02J086783 | Promoter/Enhancer | 87009725 | 87022878 | |
| cg13681325 | CD8 | *CD8A* | 2 | 87020377 | 1 | GH02J086783 | Promoter/Enhancer | 87009725 | 87022878 | |
| cg13803976 | CD8 | *CD8A* | 2 | 87016129 | 1 | GH02J086783 | Promoter/Enhancer | 87009725 | 87022878 | |
| cg13946520 | CD8 | *CD8A* | 2 | 87017689 | 1 | GH02J086783 | Promoter/Enhancer | 87009725 | 87022878 | |
| cg15195030 | CD8 | *CD8A* | 2 | 87019480 | 1 | GH02J086783 | Promoter/Enhancer | 87009725 | 87022878 | |
| cg17108819 | CD8 | *CD8A* | 2 | 87017953 | 1 | GH02J086783 | Promoter/Enhancer | 87009725 | 87022878 | |
| cg18174654 | CD8 | *CD8A* | 2 | 87019612 | 1 | GH02J086783 | Promoter/Enhancer | 87009725 | 87022878 | |
| cg18830527 | CD8 | *CD8A* | 2 | 87018966 | 1 | GH02J086783 | Promoter/Enhancer | 87009725 | 87022878 | |
| cg21116934 | CD8 | *CD8A* | 2 | 87034663 | 1 | GH02J086805 | Promoter/Enhancer | 87032335 | 87037723 | |
| cg21578555 | CD8 | *CD8A* | 2 | 87018101 | 1 | GH02J086783 | Promoter/Enhancer | 87009725 | 87022878 | |
| cg21648425 | CD8 | *CD8A* | 2 | 87019192 | 1 | GH02J086783 | Promoter/Enhancer | 87009725 | 87022878 | |
| cg22728534 | CD8 | *CD8A* | 2 | 87034800 | 1 | GH02J086805 | Promoter/Enhancer | 87032335 | 87037723 | |
| cg24345747 | CD8 | *CD8A* | 2 | 87015813 | 1 | GH02J086783 | Promoter/Enhancer | 87009725 | 87022878 | |
| cg25355010 | CD8 | *CD8A* | 2 | 87018929 | 1 | GH02J086783 | Promoter/Enhancer | 87009725 | 87022878 | |
| cg25939861 | CD8 | *CD8A* | 2 | 87020937 | 1 | GH02J086783 | Promoter/Enhancer | 87009725 | 87022878 | |
| cg26057751 | CD8 | *CD8A* | 2 | 87016259 | 1 | GH02J086783 | Promoter/Enhancer | 87009725 | 87022878 | |
| cg26730653 | CD8 | *CD8A* | 2 | 87022189 | 1 | GH02J086783 | Promoter/Enhancer | 87009725 | 87022878 | |
| cg27247697 | CD8 | *CD8A* | 2 | 87018054 | 1 | GH02J086783 | Promoter/Enhancer | 87009725 | 87022878 | |
| cg27502457 | CD8 | *CD8A* | 2 | 87018585 | 1 | GH02J086783 | Promoter/Enhancer | 87009725 | 87022878 | |
| cg01963696 | ELANE | *ELANE* | 12 | 851650 | 1 | GH19J000851 | Enhancer | 851581 | 851694 | |
| cg02487452 | ELANE | *ELANE* | 12 | 855899 | 0 |  |  |  |  | |
| cg03526702 | ELANE | *ELANE* | 12 | 856145 | 1 | GH19J000860 | Enhancer | 856001 | 856200 | |
| cg04382396 | ELANE | *ELANE* | 12 | 852311 | 1 | GH19J000852 | Promoter | 852200 | 852400 | |
| cg06100973 | ELANE | *ELANE* | 12 | 852114 | 0 |  |  |  |  | |
| cg06406619 | ELANE | *ELANE* | 12 | 851045 | 1 | GH19J000850 | Promoter/Enhancer | 850111 | 851521 | |
| cg07239938 | ELANE | *ELANE* | 12 | 852813 | 0 |  |  |  |  | |
| cg08223924 | ELANE | *ELANE* | 12 | 855922 | 0 |  |  |  |  | |
| cg08269974 | ELANE | *ELANE* | 12 | 853054 | 0 |  |  |  |  | |
| cg10393581 | ELANE | *ELANE* | 12 | 852288 | 1 | GH19J000852 | Promoter | 852200 | 852400 | |
| cg11683663 | ELANE | *ELANE* | 12 | 855536 | 0 |  |  |  |  | |
| cg12334928 | ELANE | *ELANE* | 12 | 855684 | 0 |  |  |  |  | |
| cg02082462 | PRTN3 | *PRTN3* | 12 | 848001 | 1 | GH19J000847 | Enhancer | 847402 | 848599 | |
| cg03004350 | PRTN3 | *PRTN3* | 12 | 847943 | 1 | GH19J000847 | Enhancer | 847402 | 848599 | |
| cg05257528 | PRTN3 | *PRTN3* | 12 | 846179 | 0 |  |  |  |  | |
| cg06040319 | PRTN3 | *PRTN3* | 12 | 846266 | 0 |  |  |  |  | |
| cg09134726 | PRTN3 | *PRTN3* | 12 | 841082 | 0 |  |  |  |  | |
| cg10599438 | PRTN3 | *PRTN3* | 12 | 844589 | 0 |  |  |  |  | |
| cg00473501 | MPO | *MPO* | 17 | 56354594 | 1 | GH17J058274 | Promoter/Enhancer | 56351607 | 56357412 | |
| cg02668773 | MPO | *MPO* | 17 | 56356963 | 1 | GH17J058274 | Promoter/Enhancer | 56351607 | 56357412 | |
| cg04266202 | MPO | *MPO* | 17 | 56352895 | 1 | GH17J058274 | Promoter/Enhancer | 56351607 | 56357412 | |
| cg07110356 | MPO | *MPO* | 17 | 56355431 | 1 | GH17J058274 | Promoter/Enhancer | 56351607 | 56357412 | |
| cg08058156 | MPO | *MPO* | 17 | 56356852 | 1 | GH17J058274 | Promoter/Enhancer | 56351607 | 56357412 | |
| cg09421562 | MPO | *MPO* | 17 | 56357994 | 0 |  |  |  |  | |
| cg11151395 | MPO | *MPO* | 17 | 56355299 | 1 | GH17J058274 | Promoter/Enhancer | 56351607 | 56357412 | |
| cg13842661 | MPO | *MPO* | 17 | 56352875 | 1 | GH17J058274 | Promoter/Enhancer | 56351607 | 56357412 | |
| cg14619064 | MPO | *MPO* | 17 | 56355331 | 1 | GH17J058274 | Promoter/Enhancer | 56351607 | 56357412 | |
| cg22331200 | MPO | *MPO* | 17 | 56355362 | 1 | GH17J058274 | Promoter/Enhancer | 56351607 | 56357412 | |
| cg24499524 | MPO | *MPO* | 17 | 56356470 | 1 | GH17J058274 | Promoter/Enhancer | 56351607 | 56357412 | |
| cg27456487 | MPO | *MPO* | 17 | 56349062 | 0 |  |  |  |  | |
| cg02343503 | CD14 | *CD14* | 5 | 140012023 | 1 | GH05J140630 | Promoter/Enhancer | 140009912 | 140013586 | |
| cg03251293 | CD14 | *CD14* | 5 | 140011317 | 1 | GH05J140630 | Promoter/Enhancer | 140009912 | 140013586 | |
| cg12143439 | CD14 | *CD14* | 5 | 140012660 | 1 | GH05J140630 | Promoter/Enhancer | 140009912 | 140013586 | |
| cg12192566 | CD14 | *CD14* | 5 | 140011377 | 1 | GH05J140630 | Promoter/Enhancer | 140009912 | 140013586 | |
| cg14958663 | CD14 | *CD14* | 5 | 140011662 | 1 | GH05J140630 | Promoter/Enhancer | 140009912 | 140013586 | |
| cg19008097 | CD14 | *CD14* | 5 | 140012986 | 1 | GH05J140630 | Promoter/Enhancer | 140009912 | 140013586 | |
| cg20943084 | CD14 | *CD14* | 5 | 140012769 | 1 | GH05J140630 | Promoter/Enhancer | 140009912 | 140013586 | |
| cg25358289 | CD14 | *CD14* | 5 | 140012728 | 1 | GH05J140630 | Promoter/Enhancer | 140009912 | 140013586 | |
| cg03341697 | CD15 | *FUT4* | 11 | 94277138 | 1 | GH11J094542 | Promoter/Enhancer | 94276001 | 94282967 | |
| cg12631737 | CD15 | *FUT4* | 11 | 94277190 | 1 | GH11J094542 | Promoter/Enhancer | 94276001 | 94282967 | |
| cg16095615 | CD15 | *FUT4* | 11 | 94277451 | 1 | GH11J094542 | Promoter/Enhancer | 94276001 | 94282967 | |
| cg03543495 | CD15 | *FUT4* | 11 | 94277826 | 1 | GH11J094542 | Promoter/Enhancer | 94276001 | 94282967 | |
| cg06526620 | CD15 | *FUT4* | 11 | 94278324 | 1 | GH11J094542 | Promoter/Enhancer | 94276001 | 94282967 | |
| cg05229803 | CD15 | *FUT4* | 11 | 94278407 | 1 | GH11J094542 | Promoter/Enhancer | 94276001 | 94282967 | |
| cg08863777 | CD15 | *FUT4* | 11 | 94278457 | 1 | GH11J094542 | Promoter/Enhancer | 94276001 | 94282967 | |
| cg20533957 | CD15 | *FUT4* | 11 | 94278538 | 1 | GH11J094542 | Promoter/Enhancer | 94276001 | 94282967 | |
| cg07235053 | CD15 | *FUT4* | 11 | 94278581 | 1 | GH11J094542 | Promoter/Enhancer | 94276001 | 94282967 | |
| cg04757806 | CD15 | *FUT4* | 11 | 94278595 | 1 | GH11J094542 | Promoter/Enhancer | 94276001 | 94282967 | |
| cg18023065 | CD15 | *FUT4* | 11 | 94278603 | 1 | GH11J094542 | Promoter/Enhancer | 94276001 | 94282967 | |
| cg10283505 | CD15 | *FUT4* | 11 | 94278912 | 1 | GH11J094542 | Promoter/Enhancer | 94276001 | 94282967 | |
| cg13300301 | CD15 | *FUT4* | 11 | 94279068 | 1 | GH11J094542 | Promoter/Enhancer | 94276001 | 94282967 | |
| cg16354404 | CD15 | *FUT4* | 11 | 94280121 | 1 | GH11J094542 | Promoter/Enhancer | 94276001 | 94282967 | |
| cg01335180 | CD16 | *FCGR3A* | 1 | 161576580 | 0 |  |  |  |  | |
| cg02106043 | CD16 | *FCGR3A* | 1 | 161575319 | 0 |  |  |  |  | |
| cg02403069 | CD16 | *FCGR3A* | 1 | 161520639 | 0 |  |  |  |  | |
| cg02516018 | CD16 | *FCGR3A* | 1 | 161536262 | 1 | GH01J161566 | Promoter/Enhancer | 161535790 | 161536391 | |
| cg02832305 | CD16 | *FCGR3A* | 1 | 161563355 | 0 |  |  |  |  | |
| cg03105929 | CD16 | *FCGR3A* | 1 | 161591659 | 1 | GH01J161620 | Promoter/Enhancer | 161590390 | 161595147 | |
| cg03228353 | CD16 | *FCGR3A* | 1 | 161520094 | 1 | GH01J161549 | Promoter/Enhancer | 161519536 | 161520191 | |
| cg04094791 | CD16 | *FCGR3A* | 1 | 161576629 | 0 |  |  |  |  | |
| cg04384208 | CD16 | *FCGR3A* | 1 | 161519396 | 0 |  |  |  |  | |
| cg04567009 | CD16 | *FCGR3A* | 1 | 161600769 | 0 |  |  |  |  | |
| cg05178502 | CD16 | *FCGR3A* | 1 | 161575766 | 0 |  |  |  |  | |
| cg06960562 | CD16 | *FCGR3A* | 1 | 161582601 | 1 | GH01J161611 | Promoter/Enhancer | 161581172 | 161583291 | |
| cg07111566 | CD16 | *FCGR3A* | 1 | 161516128 | 0 |  |  |  |  | |
| cg07793789 | CD16 | *FCGR3A* | 1 | 161582376 | 1 | GH01J161611 | Promoter/Enhancer | 161581172 | 161583291 | |
| cg08879849 | CD16 | *FCGR3A* | 1 | 161572679 | 0 |  |  |  |  | |
| cg08926056 | CD16 | *FCGR3A* | 1 | 161591591 | 1 | GH01J161620 | Promoter/Enhancer | 161590390 | 161595147 | |
| cg09948955 | CD16 | *FCGR3A* | 1 | 161520652 | 0 |  |  |  |  | |
| cg10073693 | CD16 | *FCGR3A* | 1 | 161590955 | 1 | GH01J161620 | Promoter/Enhancer | 161590390 | 161595147 | |
| cg10815343 | CD16 | *FCGR3A* | 1 | 161575992 | 0 |  |  |  |  | |
| cg11073684 | CD16 | *FCGR3A* | 1 | 161565284 | 0 |  |  |  |  | |
| cg11674865 | CD16 | *FCGR3A* | 1 | 161591488 | 1 | GH01J161620 | Promoter/Enhancer | 161590390 | 161595147 | |
| cg13048485 | CD16 | *FCGR3A* | 1 | 161586839 | 0 |  |  |  |  | |
| cg13139730 | CD16 | *FCGR3A* | 1 | 161576322 | 0 |  |  |  |  | |
| cg13495918 | CD16 | *FCGR3A* | 1 | 161575324 | 0 |  |  |  |  | |
| cg14534021 | CD16 | *FCGR3A* | 1 | 161561150 | 0 |  |  |  |  | |
| cg14838427 | CD16 | *FCGR3A* | 1 | 161592001 | 1 | GH01J161620 | Promoter/Enhancer | 161590390 | 161595147 | |
| cg17508302 | CD16 | *FCGR3A* | 1 | 161575716 | 0 |  |  |  |  | |
| cg18222653 | CD16 | *FCGR3A* | 1 | 161580444 | 0 |  |  |  |  | |
| cg18298815 | CD16 | *FCGR3A* | 1 | 161519989 | 1 | GH01J161549 | Promoter/Enhancer | 161519536 | 161520191 | |
| cg19242610 | CD16 | *FCGR3A* | 1 | 161569954 | 0 |  |  |  |  | |
| cg21546996 | CD16 | *FCGR3A* | 1 | 161591215 | 1 | GH01J161620 | Promoter/Enhancer | 161590390 | 161595147 | |
| cg23270415 | CD16 | *FCGR3A* | 1 | 161574664 | 0 |  |  |  |  | |
| cg24052263 | CD16 | *FCGR3A* | 1 | 161575010 | 0 |  |  |  |  | |
| cg26156167 | CD16 | *FCGR3A* | 1 | 161582651 | 1 | GH01J161611 | Promoter/Enhancer | 161581172 | 161583291 | |
| cg26186545 | CD16 | *FCGR3A* | 1 | 161520972 | 0 |  |  |  |  | |
| cg26435281 | CD16 | *FCGR3A* | 1 | 161594100 | 1 | GH01J161620 | Promoter/Enhancer | 161590390 | 161595147 | |
| cg26911986 | CD16 | *FCGR3A* | 1 | 161582046 | 1 | GH01J161611 | Promoter/Enhancer | 161581172 | 161583291 | |
| cg27514565 | CD16 | *FCGR3A* | 1 | 161581262 | 1 | GH01J161611 | Promoter/Enhancer | 161581172 | | 161583291 |
| Note: POS = position. reg. region = regulatory region. All genomic locations are displayed according to hg19. ^1^ GHID: GeneHancer identifiers. The id begins with GH, followed by the chromosome number, a single letter related to the GeneHancer version (constant since version 4.8, ‘J’), and approximate kilobase start coordinate. The data on information on regulatory regions were downloaded from Gene Cards. The human gene database. ² refers to the respective regulatory region. | | | | | | | | | | |

**Table S2.** Sequences of primers used for gene expression analyses.

| **Target gene** | **Forward primer (5´-3´)** | **Reverse primer (5´-3´)** |
| --- | --- | --- |
| *CD19* | GGCCCGAGGAACCTCTAGT | TAAGAAGGGTTTAAGCGGGGA |
| *CD8A* | ATGGCCTTACCAGTGACCG | AGGTTCCAGGTCCGATCCAG |
| *CD4* | TGCCTCAGTATGCTGGCTCT | GAGACCTTTGCCTCCTTGTTC |
| *FCGR3A(CD16)* | CCTCCTGTCTAGTCGGTTTGG | TCGAGCACCCTGTACCATTGA |
| *CD14* | ACGCCAGAACCTTGTGAGC | GCATGGATCTCCACCTCTACTG |
| *FUT4 (CD15)* | GATCTGCGCGTGTTGGACTA | GAGGGCGACTCGAAGTTCAT |
| *MPO* | TGCTGCCCTTTGACAACCTG | TGCTCCCGAAGTAAGAGGGT |
| *18s* | TGCCCTATCAACTTTCGATG | GATGTGGTAGCCGTTTCTCA |

**Statistical analyses** - Normal distribution of data (Kolmogorov-Smirnov tests):

*Immune cell type proportions:* Normal distribution of the data was confirmed: *D*(27) = 0.102 – 0.165; *p* = .056 - .200

T*arget gene associated DNA methylation:* The beta values of CD8A did not follow a normal distribution: *D*(29) = 0.165, *p* = .042

T*arget gene expression*: *CD19, CD4, FCGR3A, CD14, FUT4* and *MPO* expression data were not normally distributed: *D*(26) = 0.193 – 0.252, *p* = .014 - <.001

**Table S3.** Mean predicted proportions of leukocyte subsets and NCU vs. CCU group comparisons.

| **Cell type** | **NCU group**  **(*n* = 15)** | | **CCU group**  **(*n* = 14)** | | **Group comparison** | | |
| --- | --- | --- | --- | --- | --- | --- | --- |
|  | *M (%)* | *SD* | *M (%)* | *SD* | *t (df)* | *p_adjusted_^2^* | *d* |
| *B cells^1^* | 9.01 | 1.63 | 6.72 | 1.68 | 3.66 (26) | .007* | 1.383 |
| *CD8^+^ T cells* | 10.64 | 3.67 | 9.39 | 3.85 | 0.90 (27) | .454 | 0.333 |
| *CD4^+^ T cells* | 19.33 | 4.90 | 15.36 | 7.27 | 1.73 (27) | .189 | 0.644 |
| *NK cells^1^* | 5.76 | 4.02 | 3.84 | 3.06 | 1.42 (26) | .250 | 0.538 |
| *monocytes* | 7.76 | 2.08 | 8.27 | 1.70 | -0.71 (27) | .485 | -0.263 |
| *granulocytes* | 50.21 | 7.00 | 58.70 | 10.30 | -2.61 (27) | .044* | -0.971 |

*Note: ^1^ n* = 1 sample of the NCU group was identified as outlier, the respective value was excluded from analysis*.* ^2^ = adjusted *p*-value using the False Discovery Rate (FDR). NCU: non-cannabis-using; CCU: chronic cannabis use. Statistics: Independent *t*-tests, **p* < .05.

**Figure S1**


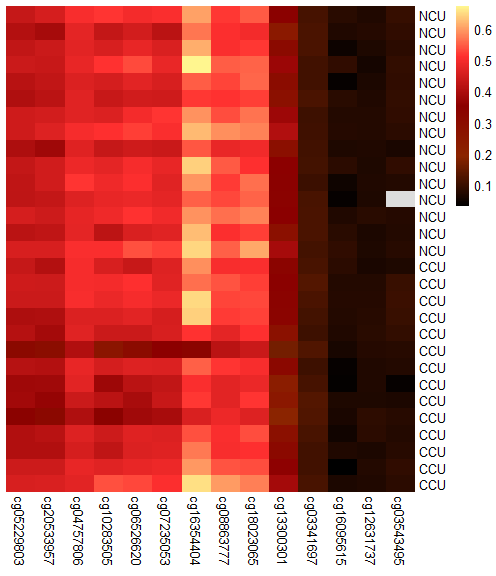

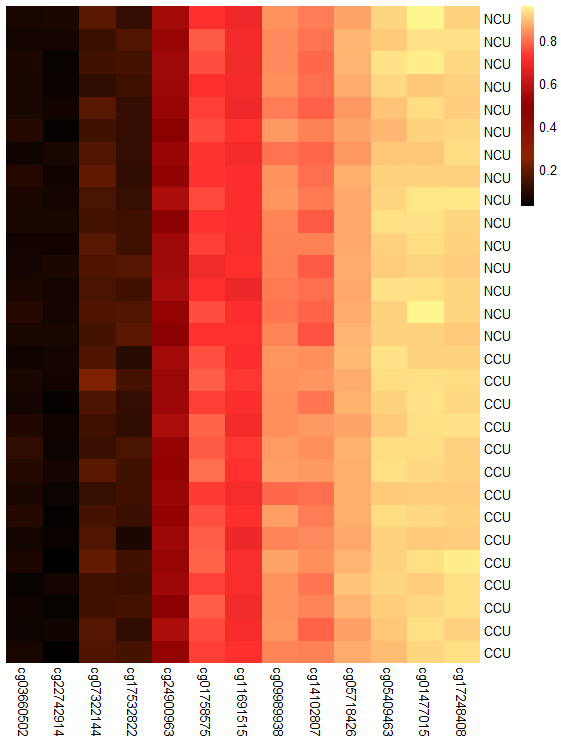
**A) B)**

**Fig. S1.** Heatmaps visualizing the methylation level (beta values, ranging from 0 to 1) of each CpG site analyzed for **A)** *CD19* and **B)** *FUT4* per sample. NCU: non-cannabis-using (*n* = 15); CCU: chronic cannabis use (*n* = 14).


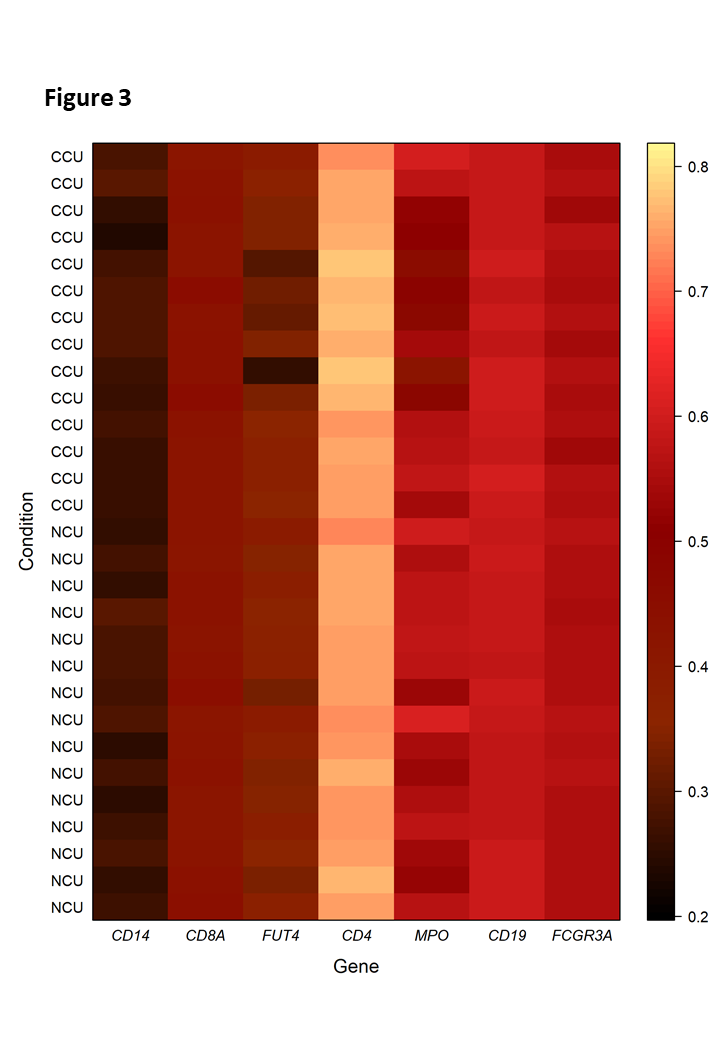
**Figure S2**

**Fig. S2.** Heatmap visualizing the mean methylation levels (beta values, ranging from 0 to 1) of all CpG sites analyzed per target gene (*k* = 7) per sample. NCU: non-cannabis-using (*n* = 15); CCU: chronic cannabis use (*n* = 14).
